# Effects of Different Sediment Improvers on the Growth Environment, Innate Immune Responses, and Intestinal Health of *Procambarus clarkii*

**DOI:** 10.3390/biology14040407

**Published:** 2025-04-11

**Authors:** Xinyu Wu, Hao Wu, Yifan Wu, Zhiqiang Xu, Hong Shan, Tianheng Gao

**Affiliations:** 1 College of Oceanography, Hohai University, Nanjing 210024, China; 221311040020@hhu.edu.cn (X.W.); 211311040023@hhu.edu.cn (H.W.); 221611010017@hhu.edu.cn (Y.W.); 2 Freshwater Fisheries Research Institute of Jiangsu Province, Nanjing 210017, China; zhiqiangx@163.com; 3 Nanjing Institute of Fisheries Science, Nanjing 210017, China; 4 College of Marine Science and Engineering, Nanjing Normal University, Nanjing 210023, China

**Keywords:** sediment improvers, *Procambarus clarkii*, water quality, immunity, intestinal microbiota

## Abstract

Sediment improvers are important mediators of aquatic animals’ growth performance and the surrounding environmental quality. However, the physiological responses of crayfish (*Procambarus clarkii*) to different sediment improvers remain unclear. Here, we cultivated crayfish using two chemical (potassium monopersulfate and potassium ferrate) and two biological (purple nonsulfur photosynthetic bacteria and *Bacillus subtilis*) sediment improvers at low and high concentrations. After 42 days, we found that the addition of chemical sediment improvers was more effective in improving water quality than biological sediment improvers (e.g., more stable pH and lower nutrient concentrations). By contrast, the application of biological sediment improvers resulted in considerably enhanced final weight, weight gains, and survival rates. In all low-concentration groups, the activity of immune-related enzymes (e.g., superoxide dismutase and glutathione peroxidase) in the hemolymph and hepatopancreas considerably increased, whereas the malondialdehyde activity and mRNA expression of AMP genes (*PcALF* and *PcCru*) considerably decreased. Crayfish exposed to low concentrations of sediment improvers exhibited enhanced intestinal and hepatopancreatic integrity, with a thickened mucosal layer and increased density of epithelial cell granules. Additionally, the composition of the gut microbiota varied after the addition of different sediment improvers. In summary, our research indicated that different types of sediment improvers not only improved the farming environment but also had varying effects on crayfish. Therefore, an appropriate sediment improver based on specific aquaculture conditions is needed.

## Simple Summary

Sediment improvers are important mediators of aquatic animals’ growth performance and the surrounding environmental quality. However, the physiological responses of crayfish (*Procambarus clarkii*) to different sediment improvers remain unclear. Here, we cultivated crayfish using two chemical (potassium monopersulfate and potassium ferrate) and two biological (purple nonsulfur photosynthetic bacteria and *Bacillus subtilis*) sediment improvers at low and high concentrations. After 42 days, we found that the addition of chemical sediment improvers were more effective in improving water quality than biological sediment improvers. By contrast, the application of biological sediment improvers resulted in considerably enhanced final weight, weight gains, and survival rates. In all low-concentration groups, the activity of immune-related enzymes in the hemolymph and hepatopancreas considerably increased, whereas the malondialdehyde activity and mRNA expression of AMP genes (PcALF and PcCru) considerably decreased. Crayfish exposed to low concentrations of sediment improvers exhibited enhanced intestinal and hepatopancreatic integrity. Additionally, the composition of the gut microbiota varied after the addition of different sediment improvers. In summary, our research indicated that different types of sediment improvers not only improved the farming environment but also had varying effects on crayfish. Therefore, an appropriate sediment improver based on specific aquaculture conditions is needed.

## 1. Introduction

Aquaculture is a crucial component of the global food industry, contributing considerably to global food security and development [1]. According to the State of World Fisheries and Aquaculture 2024 report by the Food and Agriculture Organization of the United Nations (FAO), global fisheries and aquaculture production surged to 223.2 million tonnes in 2022, an increase of 4.4% over 2020 (World Food Network: http://www.shijieshipin.com, accessed 8 March 2025). By 2022, the total output value of China’s crayfish industry climbed to $64.07 billion, an increase of 7.99% year on year, which was an improvement from the growth rate of $59.33 billion in 2021. In the same year, China’s crayfish farming area expanded to 18.67 million hectares, with a yield of 2.89 million tons, increasing at an average annual rate of 7.69% and 9.76%, respectively. Its production accounts for 8.79% of the total freshwater aquaculture production, making the crayfish the fourth largest freshwater aquaculture species in China [2]. Thus, the crayfish plays an important role in promoting the high-quality development of the fishery industry and advancing rural revitalization (China Crayfish Industry Report, http://www.nftec.agri.cn/, accessed on 8 March 2025). The crayfish exhibits specific habitat preferences and behaviors. It is a benthic organism that exhibits burrowing behavior [3] and prefers muddy or sandy substrates in shallow waters, where it can construct burrows to protect itself from predators and environmental stressors [4]. Thus, sediment substrates are of paramount importance for the survival of crayfish in aquatic environments.

In response to the increasing market demand, farmers frequently increase the stocking density of crayfish to maximize profits and meet consumer needs. However, this practice exerts considerable pressure on the health and growth of crayfish [5]. Intensive farming conditions can result in the accumulation of shrimp excrement, dead biomass, organic fertilizers, and residual feed at the pond bottom, particularly during the mid-to-late stages of farming [6]. Notably, the accumulation of waste can detrimentally affect the growth and development of cultured shrimp and crabs [7]. Consequently, removing these waste products and improving the quality of pond substrates are essential to aquaculture management [8].

In aquaculture, sediment improvers are important agents for improving water quality and reducing harmful compounds, such as ammonia nitrogen, nitrite, and sulfides, which can have detrimental effects on aquatic ecosystems [9]. With the expansion of global intensive aquaculture areas, the application of sediment improvers in aquaculture ponds has become increasingly common [10]. The aim of adding sediment improvers is to enhance water suitability for aquatic organisms and thereby promote their growth and reproduction. In recent years, the most commonly utilized sediment improvers include activated carbon, potassium monopersulfate (KMPS), *Bacillus subtilis* (BS), and an array of composite bacterial agents [11]. Another concern in aquaculture is microbial pollution, particularly the prevalence of blue-green algae, which has a deleterious effect on the growth of cultivated species [12]. Ferrate is an effective agent for inactivating common Cyanobacteria, including *Microcystis* and *Anabaena* and the toxic microcystin MC-LR [13].

In aquaculture, sediment improvers, such as KMPS, potassium ferrate (PF), purple nonsulfur photosynthetic bacteria (PNSBs), and BS, offer many benefits. KMPS reduces ammonia nitrogen levels in shrimp farming systems, the number of culturable bacteria, and the proportion of *Vibrio* in shrimp and water [11,14]. Moreover, KMPS enhances water quality, immune response, and growth performance in the short term [15]. In GIFT tilapia cultivation, BS improves water quality, growth performance, immune levels, antioxidant capacity, and immune response [16] *Bacillus* species confer benefits to the microbial community and to the development of Pacific white shrimp (*Litopenaeus vannamei*), increasing survival rates, enhancing immune gene expression, and heightening resistance to *Vibrio harveyi* infection [17]. The positive effects of PNSB in aquaculture applications have been documented [18]. Adding PNSB to feed improves shrimp growth performance, protein digestion activity, and antioxidant capacity [19]. In conclusion, sediment improvers, such as KMPS, PF, PNSB, and BS, play a crucial role in enhancing the growth of aquatic animals by inhibiting pathogenic microorganisms, boosting host immunity, and promoting growth factors.

However, most studies have primarily analyzed the bactericidal and disinfectant properties of specific sediment improvers, leaving a gap regarding their effects on aquaculture species, particularly for crayfish. The purpose of this study was to explore the effects of sediment amendments on the culture environment of crayfish, with particular attention to how different types and concentrations of sediment amendments affect the growth environment, innate immunity, and intestinal microbial community of crayfish. We hypothesized that low concentrations of chemical sediment modifiers KMPS and PF can significantly improve water quality and environmental conditions, while low concentrations of the biological sediment modifiers PNSB and BS can enhance the innate immunoenzyme activity and antimicrobial peptide gene expression of crayfish. Further, we speculate that different types of sediment amendments will have different biological effects on crayfish and that high concentrations of sediment amendments may have negative effects. This study innovatively and simultaneously evaluated the effects of different sediment amendments on various aspects of crayfish, aiming to provide theoretical support for optimizing their use strategies and promoting the sustainable development of the aquaculture industry.

## 2. Materials and Methods

### 2.1. Experimental Crayfish and Sediment Improvers

A total of 540 living crayfish (13.84 ± 0.58 g) were collected from a farming pond in Huai’an, Jiangsu Province, China. Before the experiment, the crayfish were temporarily cultured in 70 cm × 70 cm × 50 cm glass tanks for two weeks. The feeding water was tap water boiled for more than two days, the water temperature was maintained between 20 °C and 25 °C, the pH was approximately 7.5, and dissolved oxygen (DO) levels were over 5.0 mg/L. The water was changed every three days, and the crayfish were fed with artificial feed at a rate of 1–3% of their body weight. Feeding was stopped 24 h before the experiment. Healthy, active, and disease-free crayfish of uniform size were selected for the experiment. KMPS and PF were purchased from Shanghai Aladdin Biochemical Technology Co., Ltd. (Shanghai, China). PNSB and BS were purchased from Jiangsu Zunsheng Rong Agricultural Service Co., Ltd. (Nanjing, China). Artificial feed was purchased from Tongwei Co., Ltd. (Chengdu, China).

### 2.2. Experimental Design and Feeding Management

Healthy crayfish were divided into nine groups, with three replicates for each group, and raised in 300 L aquariums (60 crayfish per group): CK (control), KL (0.5 mg/L of KMPS), KH (5 mg/L of KMPS), FL (0.5 mg/L of PF), FH (5 mg/L of PF), PL (1 × 10^6^ CFU/mL PNSB), PH (1 × 10^7^ CFU/mL PNSB), BL (1 × 10^6^ CFU/mL BS), and BH (1 × 10^7^ CFU/mL BS) [20,21]. These concentrations represent the minimum and maximum acceptable concentrations of sediment amendments for the crayfish. Sediment improvers were applied every 14 days during the breeding period. Prior to application, the mixture was dissolved in water and then evenly distributed into the culture water and thoroughly mixed. During cultivation, the crayfish were fed with commercial feed twice daily at 8:00 and 18:00, and the amount was adjusted according to their feeding and activity levels. Crayfish mortality was determined by the cessation of gill movement, and dead crayfish were promptly removed. No biological filtration system was used in this experiment.

### 2.3. Determination of Growth Performance

The growth performance of the crayfish was calculated according to the change between the beginning and end of the experiment, with the following formulas:

Weight gain (WG) = (Wt − Wo)/Wo × 100

Survival rate = Nt/No × 100

where Wt and Wo are the final and initial mean individual weights of the crayfish in three replicates, and Nt and No are the survival number and initial total number of the crayfish in each group.

### 2.4. Sample Collection

The crayfish samples were collected 42 days after feeding followed by 24 h of starvation. The hemolymph (0.1 mL) and hepatopancreas (0.1 g) were collected from three crayfish in each replicate for enzyme activity assays and RNA extraction. The hemolymph was collected using a chilled anticoagulant buffer (0.14 M NaCl, 30 mM trisodium citrate, 26 mM citric acid, 10 mM EDTA, 0.1 M glucose, pH 4.6). The hepatopancreas and intestinal tissues were carefully sampled, fixed in formaldehyde, and subjected to histopathological examination. The crayfish were then disinfected with 75% ethanol before dissection, and intestinal samples were collected. The intestinal contents were scraped, snap-frozen in liquid nitrogen, and stored at −80 °C for subsequent microbial DNA extraction.

### 2.5. Determination of Water Quality

Throughout the entire experiment, water quality was measured in terms of water pH and DO, ammonia nitrogen, nitrite, total nitrogen (TN), and total phosphorus (TP) levels. Each measurement was taken once on the last day of the week. The pH and DO levels were measured using a water quality analyzer (Shanghai Instrument and Electronics Science Instrument Co., Ltd., Shanghai, China), and the concentrations of ammonia nitrogen, nitrite, TN, and TP were determined using spectrophotometric methods.

### 2.6. Determination of Immune Enzyme Activity

Proteins were extracted from the hepatopancreas and hemolymph, total protein (TP) concentration was determined, and the enzyme activity of glutathione peroxidase (GSH-PX), total superoxide dismutase (T-SOD), acid phosphatase (ACP), alkaline phosphatase (AKP), and malondialdehyde (MDA) was measured. Enzyme activity was assessed using commercial assay kits purchased from Jiancheng Bioengineering Institute (NJJCbio, Nanjing, China).

### 2.7. Detection of Immune-Related Gene Expression

Total RNA was extracted from the hepatopancreas and hemolymph with a commercial reagent kit (RNAiso Plus, TaKaRa, Dalian, China) according to the manufacturer’s instructions. Subsequently, 1 μg of total RNA was used in synthesizing the first-strand cDNA with PrimeScript RT Master Mix (Perfect Real Time, TaKaRa, Dalian, China). The expression levels of antimicrobial peptides (*PcALF1*, *PcALF4*, *PcALF12*, *PcCru3*, *PcCru6*, and *PcCru10*) were determined and designed. The 18S rRNA was used as the internal reference gene. The primers used are listed in Table 1. The PCR amplification conditions were set as follows: 95 °C for 3 min, followed by 40 cycles of 95 °C for 5 s, and 60 °C for 30 s. All samples were tested in triplicate, and the results were calculated using the 2^−△△Ct^ method.

### 2.8. Histological Examination of Intestine and Hepatopancreas

The collected tissue samples were fixed in 10% formalin solution for 48 h. Subsequently, the samples were dehydrated, cleared, embedded, sectioned, stained with hematoxylin and eosin, and stained with Oil Red O with established histological techniques. Morphological changes in the tissues were observed under a light microscope.

### 2.9. 16S rRNA Gene Sequence Analysis

The intestinal contents were sent to Magigene Biotechnology Co., Ltd. (Guangzhou, China), and 16S rRNA gene sequence analysis was conducted according to our previous study. In brief, DNA was isolated, 16S rRNA was amplificated, and genomic libraries were constructed. Subsequently, an Illumina MiSeq system was used in sequencing the prepared libraries, and 16S rRNA sequencing reads were analyzed using the Quantitative Insights Into Microbial Ecology pipeline. The operational taxonomic units (OTUs) were clustered on the basis of filtered high-quality reads (similarity > 97%).

### 2.10. Statistical Analysis

Origin 2024 software was used in conducting related statistical analyses, and the data were presented as mean ± SD (*n* = 3). One-way analysis of variance (ANOVA) was employed. Lowercase letters (a, b, c, d, and e) denote significant differences among different sampling groups (determined by Tukey’s test, *p* < 0.05). Enzyme and gene bar charts were generated using GraphPad Prism 9, and microbial data visualizations were created using ChiPlot (https://www.chiplot.online/, accessed on 1 August 2024).

## 3. Results

### 3.1. Effects of Different Sediment Amendments on Water Quality

**Figure 1:**
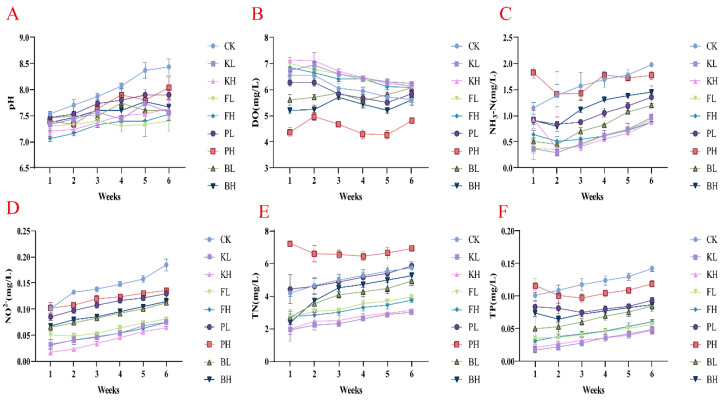
Variation in water quality in *Procambarus clarkii* aquaculture environment after treatment with different sediment improvers. (**A**) pH value; (**B**) Dissolved oxygen (DO); (**C**) Ammonia nitrogen (NH_3_-N); (**D**) Nitrite (NO_2−_); (**E**) Total nitrogen (TN); (**F**) Total phosphorus (TP).

Sediment improvers stabilized water pH (Figure 1A). The increase in pH in the KL group observed after the sixth week was only 6.67%, whereas the CK group showed an increase of 10.92%. Moreover, KMPS, PF, and BS effectively reduced the levels of ammonia nitrogen, nitrite nitrogen, TN, and TP in the water to varying degrees, and KMPS and PF showed the most considerable effects. Furthermore, KMPS and PF had increased DO levels (Figure 1C–F). Compared with the CK group, the KH group exhibited a 55.69% reduction (*p* < 0.05) in ammonia nitrogen, whereas the BH group showed a 26.56% reduction in the final week.

### 3.2. Effects of Different Sediment Amendments on Growth and Survival of Crayfish

**Figure 2:**
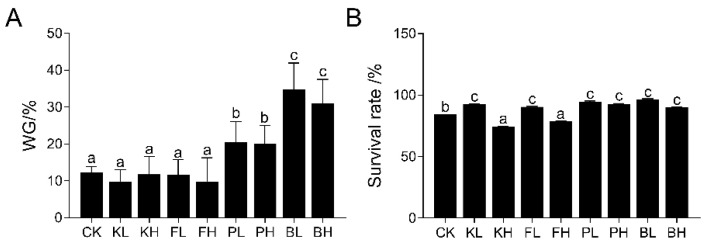
Effects of different sediment improvers on the growth performance of crayfish. (**A**) Weight gain (WG), (**B**) survival rate. Significance differences were determined by one-way analysis of variance (ANOVA), with different letters indicating significant differences. The bar chart represents mean ± standard deviation (*n* = 3).

The growth performance of crayfish during the feeding period in each group is shown in Figure 2A,B (*p* < 0.05). The final body weight of crayfish in the biological sediment improver groups was significantly higher than that in the CK group (*p* < 0.05). However, no significant differences in the final body weight of crayfish were found in the chemical sediment improver groups compared with the CK group, and the survival rates of the KH and FH groups significantly decreased. The KH and FH groups showed 11.48% and 6.67% decreases, respectively.

### 3.3. The Activity of Antioxidant Enzymes in the Blood and Hepatopancreas of Crayfish Was Affected by Sediment Amendments

**Figure 3:**
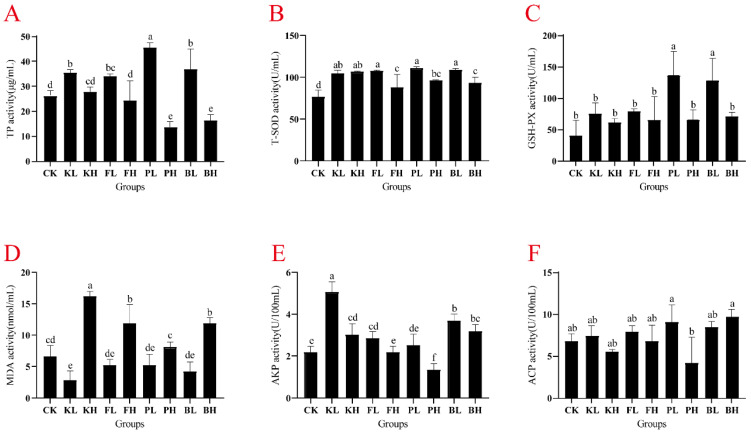
Variation in immune-related enzyme activity in the hemolymph of *Procambarus clarkii* subjected to different sediment improver treatments. (**A**) Total protein (TP), (**B**) total superoxide dismutase (T-SOD), (**C**) glutathione peroxidase (GSH-PX), (**D**) malondialdehyde (MDA), (**E**) alkaline phosphatase (AKP), and (**F**) acid phosphatase (ACP). Significant difference was determined by using one-way analysis of variance (ANOVA), and different letters indicate significant difference. Bars represent the mean ± S.D (*n* = 3).

Compared with the CK group, the concentrations of TP in the hemolymph significantly increased in the low-concentration groups, and the PL group showed the highest increase (74.12%; Figure 3A; *p* < 0.05). T-SOD activity was significantly elevated after the addition of each sediment improver (Figure 3B). Furthermore, the PL and BL groups showed significant enhancement in GSH-PX activity (Figure 3C; *p* < 0.05). After the addition of a low concentration of potassium persulfate, MDA activity significantly decreased, and MDA activity significantly increased after the addition of high concentrations of each sediment improver (Figure 3D, *p* < 0.05). In the hepatopancreas, TP content significantly increased in all experimental groups compared with the CK group (Figure S1A, *p* < 0.05). T-SOD activity was significantly elevated in the FL, PL, and BL groups (Figure S1B, *p* < 0.05). GSH-PX activity was significantly enhanced in the PL and BL groups (Figure S1C, *p* < 0.05). After sediment improvers were added, MDA activity significantly decreased (Figure S1D, *p* < 0.05). AKP activity did not show significant changes after the addition of different sediment improvers (Figure S1E). In summary, the addition of an appropriate amount of sediment improver enhanced the antioxidant capacity of crayfish to varying degrees.

### 3.4. Sediment Amendments Affect Variation in the Expression of Immune-Related Genes in Hepatopancreas and Blood

**Figure 4:**
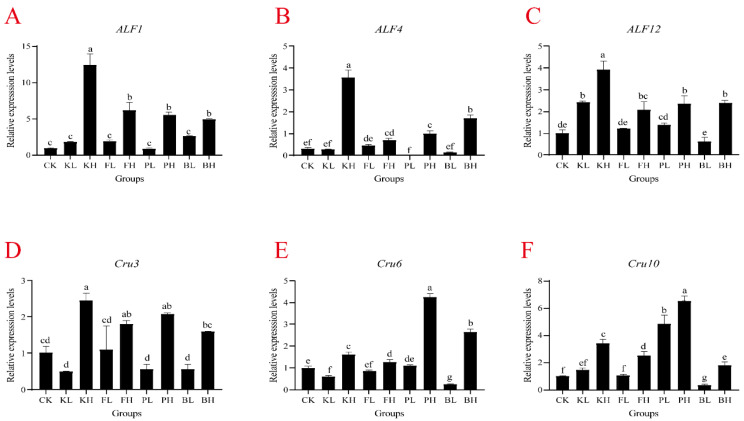
mRNA expression in the hemolymph of *Procambarus clarkii* after supplementation with sediment improvers. (**A**) *ALF1*, (**B**) *ALF4*, (**C**) *ALF12*, (**D**) *Cru3*, (**E**) *Cru6*, and (**F**) *Cru10*. One-way analysis of variance (ANOVA) was used in determining significant difference, and different letters indicate significant differences. Bars represent the mean ± S.D. (*n* = 3).

To determine whether different sediment improvers affect the expression of immune-related genes in crayfish, we selected six immune-related genes (*PcALF1*, *PcALF4*, *PcALF12*, *PcCru3*, *PcCru6*, and *PcCru10*) and measured their expression levels in the hemolymph and hepatopancreas. In the hemolymph, the expression levels of antimicrobial peptide genes significantly increased after the addition of high-concentration sediment improvers, particularly in the KH group, where the expression levels were more than four times those of the CK group. (Figure 4; *p* < 0.05). The KL and BL groups showed a significant decrease in *PcCru6* gene expression (Figure 4, *p* < 0.05), and the BS group showed significant decreases in *PcCru6* and *PcCru8* expression levels (Figure 4; *p* < 0.05). In the hepatopancreas, the expression level of the *PcALF1* gene significantly increased in the KH and FH groups compared with the CK group (Figure S2A; *p* < 0.05). *PcALF4* and *PcCru10* have similar expression patterns, and their expression levels significantly increased in the KH, FH, and PH groups (Figure S2B,F, *p* < 0.05). In the BS group, the expression levels of *PcALF12*, *PcCru6*, and *PcCru10* antimicrobial peptide genes significantly decreased (Figure S2; *p* < 0.05). Overall, an appropriate amount of sediment improver can stabilize the secretion of antimicrobial peptides in crayfish, maintaining their immune capacity, and biological sediment improvers showed relatively good effects.

### 3.5. Sediment Amendments Affect Intestinal and Hepatopancreas Morphological Changes

**Figure 5:**
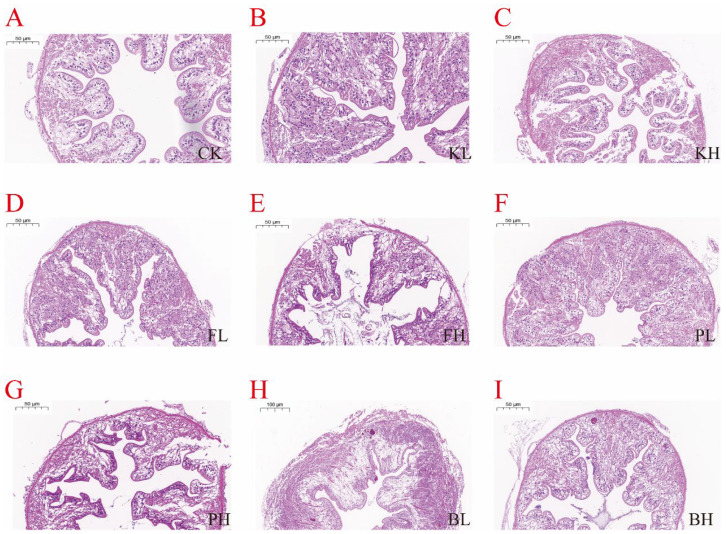
Histopathological changes in the intestines of *Procambarus clarkii* after treatment with different sediment improvers. (**A**): Control group; (**B**): low-concentration KMPS group; (**C**): high-concentration KMPS group; (**D**): low-concentration PF group; (**E**): high-concentration PF group; (**F**): low-concentration PNSB group; (**G**): high-concentration PNSB group; (**H**): low-concentration BS group; (**I**): high-concentration BS group.

**Figure 6:**
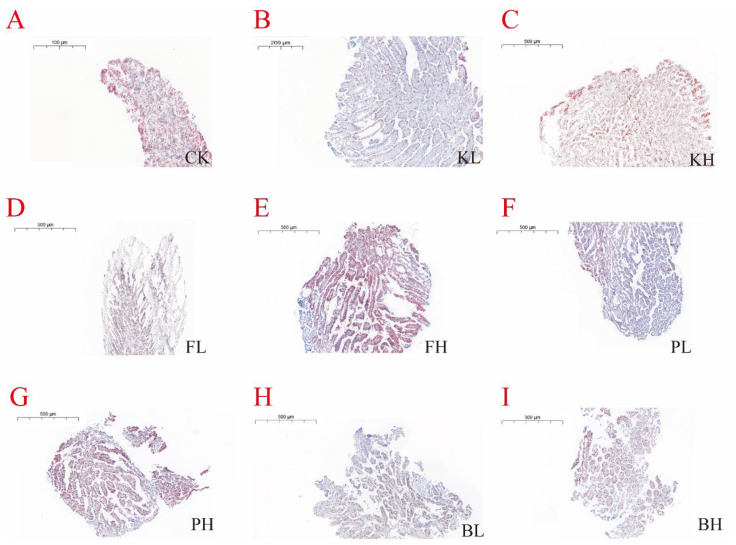
Histopathological changes in the hepatopancreas of *Procambarus clarkii* after treatment with different sediment improvers. (**A**): Control group; (**B**): low-concentration KMPS group; (**C**): high-concentration KMPS group; (**D**): low-concentration PF group; (**E**): high-concentration PF group; (**F**): low-concentration PNSB group; (**G**): high-concentration PNSB group; (**H**): low-concentration BS group; (**I**): high-concentration BS group.

Changes were observed in crayfish farming when KMPS, PF, PNSB, and BS were added. Histopathological examination of the intestinal tissues showed that the intestinal villi lengths in the low-concentration KMPS, PF, PNSB, and BS groups were uniform and longer than those in the CK group (Figure 5). Histopathological examination of the hepatopancreas tissues indicated that the low-concentration groups compared with the CK group showed reduced fat and a decreased degree of fatty liver (Figure 6). However, with the exception of the high-concentration BS group, the high-concentration groups exhibited dark coloration, indicating increased fat accumulation, high fatty liver content, and disorganized cell arrangement.

### 3.6. Sediment Amendments Alter the Composition of Intestinal Microbial Communities

**Figure 7:**
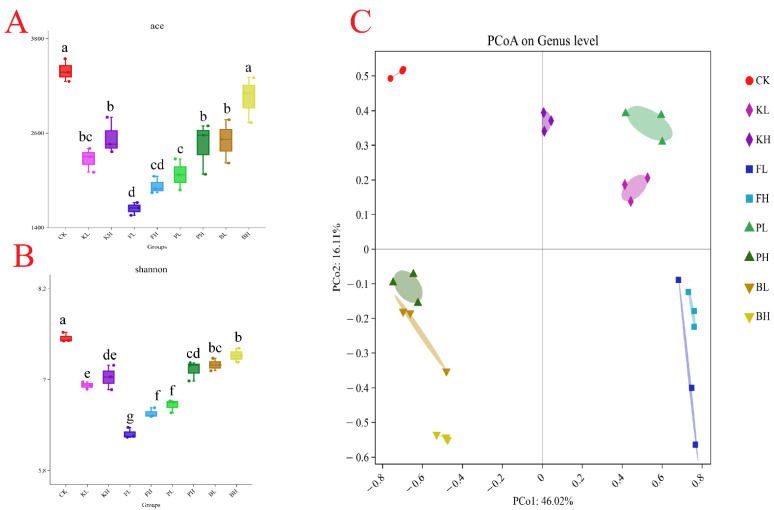
Diversity of gut microbiota in different groups. (**A**): Bacterial community diversity (measured by the ace index); (**B**): bacterial community richness (measured by the Shannon index). Different letters indicate significant differences. (**C**): Principal coordinate analysis.

**Figure 8:**
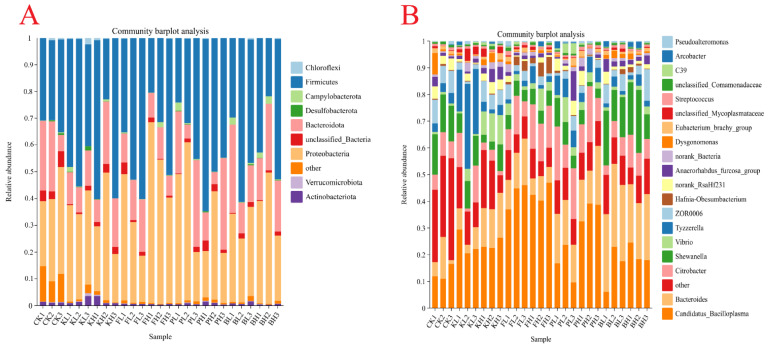
Composition of the gut microbiota in each group at the phylum (**A**) and genus levels (**B**). Taxa with abundances < 1% are included in “others”.

A total of 12,245 bacterial OTUs were identified in all the samples, and the sequencing depth exceeded 99%. High richness was observed in the CK group (Figure 7A,B). Additionally, the structure of the intestinal microbiota of crayfish considerably differed after treatment with different sediment improvers. The PCoA analysis results showed that the replicate groups were distinctly separated from one another (Figure 7C). To further understand compositional changes in the intestinal microbial communities of crayfish after the addition of sediment improvers, we performed a bar plot analysis of bacterial communities at the phylum and genus levels. The intestinal samples of untreated crayfish primarily contained *Proteobacteria*, *Firmicutes*, and *Bacteroidetes* at the phylum level. After the addition of certain sediment improvers, the abundances of *Firmicutes* and *Proteobacteria* changed, whereas the abundances of other phyla considerably decreased (Figure 8A). The above results indicated that the addition of sediment improvers altered the composition of the intestinal microbial communities of crayfish at the phylum level. Similarly, at the genus level, the composition of the intestinal microbial communities changed after the addition of sediment improvers (Figure 8B).

## 4. Discussion

A good growth environment is crucial for ensuring the healthy development of animals. Among the key toxic substances produced from aquatic animal feces and leftover feed are ammonia nitrogen and nitrite. Once these substances exceed certain thresholds, they can lead to the death of fish and shrimp [22]. Additionally, pH and DO are of great importance to crayfish growth [23]. KMPS is effective in algal and bacterial control and in improving water quality in eutrophic waters [24]. PF can remove impurities produced in aquaculture and has a certain removal efficiency for COD, ammonia nitrogen, and nitrite [25]. In grass carp aquaculture systems, adding PSB can improve water quality and alter the microbial community structure [26]. BS effectively maintains water quality and biological remediation in aquaculture, reducing organic load and thereby recycling nutrients in water and reducing sludge accumulation [27]. In this study, the chemical sediment improver groups demonstrated rapid reduction in ammonia nitrogen, nitrite, TN, and TP levels while maintaining DO content. These effects may be attributed to the direct effects of chemical improvers on harmful algae and bacteria. Biological improvers require time to occupy ecological niches. The biological improver groups initially showed a decrease in DO, likely because biological improvers consume considerable amounts of DO [28]. Overall, sediment improvers can improve water quality, and chemical sediment improvers show pronounced effects.

Animal growth performance serves as a general and intuitive indicator for assessing the effects of sediment improvers. BS can enhance growth rates because of its dual role as a sediment improver and a probiotic in aquaculture environments [29]. After the addition of three types of PSB, considerable improvements were observed in the growth of fairy shrimp (*Streptocephalus sirindhornae*) and water quality [30]. The present study demonstrated that the addition of BS and PNSB as sediment improvers had a positive effect on water quality regulation and promoted the growth and survival of crayfish. Currently, reports on the effects of KMPS and PF on aquatic organism growth are limited. However, the addition of low concentrations of KMPS and PF did not considerably affect the growth of crayfish, whereas high concentrations of KMPS and PF led to a considerable decrease in survival rates owing to the strong oxidative potential of free radicals produced in water by KMPS and PF. These free radicals, especially at high concentrations, potentially cause oxidative damage to crayfish [31].

Antioxidant capacity is an important indicator for evaluating the body’s ability to scavenge free radicals and reduce damage. An increase in a host’s endogenous antioxidant enzyme levels can enhance the animal’s immune function. [32]. For instance, PF-treated zebrafish contaminated with wastewater pollutants showed considerably reduced drug toxicity and improved antioxidant capacity [33]. The addition of KMPS, THPS, BS, and CS as water quality regulators greatly improved the antioxidant capacity of tilapia [16]. In the current study, the low-concentration groups showed a considerable increase in antioxidant enzyme activity compared with the CK group, indicating that sediment improvers can enhance the immune capability of crayfish. Additionally, the low concentrations of sediment improvers substantially reduced MDA levels, whereas high concentrations increased MDA levels in the hemolymph. MDA affects the integrity of biomembrane molecules and is used in assessing the health status of organisms. Excessive MDA levels may lead to inflammation [34]. Thus, moderate levels of sediment improvers have a positive impact on crayfish. GSH-PX and T-SOD activity, which maintain blood cell homeostasis, was considerably increased in the PL and BL groups. This result indicated that the experimental groups’ antioxidant capacity was enhanced after the addition of appropriate amounts of PSB and BS. In summary, sediment improvers at appropriate concentrations can enhance the antioxidant capacity of crayfish, and biologically active improvers showed increased efficacy.

Similar to other crustaceans, crayfish lack adaptive immunity and rely solely on innate immunity to resist diseases [35]. The interaction between the immune host and a symbiotic partner, which may release metabolites that are harmful to the host, is critical, and the host’s immune system may need to recognize and respond to these threats [36,37]. When pathogenic microorganisms breach the physical barriers of crayfish, the immune response is activated, leading to the secretion of antimicrobial peptides [38]. ALF and crustin are peptides exhibiting antibacterial activity [39]. ALF protects shrimp from bacterial and fungal infections by binding to lipopolysaccharides (LPSs) and neutralizing their activity [40]. LPSs activate inflammatory responses, leading to the production of a large amount of reactive oxygen species, which induce lipid peroxidation and MDA formation [41]. In this experiment, high concentrations of sediment improvers elevated MDA levels in the hemolymph. The expression levels of *ALF1*, *ALF4*, and *ALF12* in the high-concentration groups considerably increased. These changes might be the crustaceans’ stress response to adverse environmental conditions, leading to the secretion of large amounts of antimicrobial peptides. In the hepatopancreas, the expression levels of *ALF* remained relatively stable in the BS group, possibly because BS, as a sediment improver, does not pollute the environment. In addition, organisms developing resistance to it is unlikely [42]. Additionally, the crustin family is another crucial group of antimicrobial peptides in crustaceans and primarily exhibits bactericidal activity against Gram-positive bacteria [43]. In this experiment, high concentrations of sediment improvers led to a considerable increase in *Cru* expression, likely due to the crustaceans’ self-protection mechanisms. In the hepatopancreas, the experimental groups showed decreased *Cru3* and *Cru6* expression levels, indicating that sediment improvers improved the environment. BS remained relatively stable. Histopathological examination of the hepatopancreas revealed that the CK group treated with high concentrations of chemical sediment improvers exhibited dark coloration, which indicated increased fat accumulation, an increase in fatty liver, and disordered arrangement. By contrast, the group treated with biological sediment improvers displayed light coloration and low fatty liver content, which correlated with the expression results of antimicrobial peptides in the hepatopancreas. In conclusion, high concentrations of chemical sediment amendments may cause damage to the intestinal tract and hepatopancreas of crayfish, while moderate amounts of sediment amendments can maintain the health of these organs and have positive effects on antimicrobial peptide gene expression. BS maintained stable antimicrobial peptide expression levels in the hepatopancreas.

In crayfish, the structure of the gut microbiota can serve as an indicator of host health. The balance of the microbial community plays a crucial and beneficial role in nutrient absorption and intestinal immune response [29]. In a farming system of *Litopenaeus vannamei*, KMPS did not considerably affect the richness of the gut microbiota [15]. After BS was added as a probiotic to the diet of Nile tilapia, it improved the composition of the intestinal microbiota [44]. In this experiment, the addition of KMPS and PF led to a decrease in microbial diversity because these chemical improvers not only kill pathogens but also potentially affect beneficial bacteria, disrupting the balance of the gut microbiota.

Sediment improvers can affect the composition of the gut microbiota. The composition of the bacterial communities observed in shrimp intestines in this study indicated that *Proteobacteria* is the most dominant phylum present, followed by *Firmicutes*, *Bacteroidetes*, and *Actinobacteria*. The healthy balance of these gut microbiota is crucial for nutritional absorption, immune function, and defense against harmful pathogens in crayfish. Probiotics in the bacterial community may directly act on the immune system of crayfish, stimulating the expression of antimicrobial peptide genes, thereby enhancing defense against pathogenic microorganisms. At the same time, probiotics may also indirectly affect the expression of antibacterial genes by changing the composition of intestinal flora, reducing the stimulation of the immune system of crayfish [45]. The dysbiosis of the microbiota may lead to digestive disorders, weakened immunity, and even the onset of diseases [46] At the phylum level, sediment improvers considerably reduced the presence of other phyla, leading to the dominance of the primary phyla. This finding indicated that the sediment improvers enhance gut microbial composition. At the genus level, the relative abundance of the genus *Tyzzerella* increased after the addition of low concentrations of potassium bisulfate, which is crucial for some physiological and biochemical functions in the crustacean gut [47]. After the addition of PF, no considerable changes in the gut microbiota occurred at the genus level. However, the relative abundance of *Bacteroides* increased after the addition of PNSB and BS. This increase enhanced the competitiveness of the gut microbiota, playing a crucial role in protecting and promoting the overall health of the shrimp [48]. Histopathological analysis of intestinal sections revealed that high concentrations of chemical sediment improvers caused intestinal damage to the shrimps. The group treated with BS exhibited high-density granules within epithelial cells, which indicated active absorption and endocrine activity. In conclusion, appropriate amounts of sediment improvers, particularly biological sediment improvers, can improve the gut microbiota of crayfish.

However, there are some limitations to this study. First of all, a biological filtration system was not used in the experiment, which simulated the breeding environment of traditional farmers to a certain extent but may be different from the water quality management measures in actual farming. The absence of biofiltration systems, which are essential for maintaining stable water quality, can lead to elevated concentrations of ammonia nitrogen and nitrite, affecting the universality of experimental results. Therefore, future studies could repeat the experiment in a farming environment equipped with a biological filtration system to validate the findings of this study. Second, high concentrations of ammonium and nitrite were observed in the experiment, which may be related to improper experimental operation or inadequate water quality management. Although we observed the potential of sediment amendments to improve water quality in our experiments, these results may be influenced by water quality management. Future studies will further optimize the experimental design to reduce the impact of water quality fluctuations on the experimental results.

## 5. Conclusions

In our study, we found that adding low concentrations of KMPS and PF to water greatly improved water quality and environmental conditions, while low concentrations of PNSB and BS enhanced innate immunoenzyme activity and antimicrobial peptide gene expression in crayfish. In particular, the BS group maintained the integrity of the intestinal epithelial cells and had a thick mucus layer. Also, the epithelial cell particle density increased, indicating a strengthened intestinal barrier. In addition, the PNSB and BS groups saw improved diversity of the gut microbial community of crayfish at the genus and phylum levels, thereby enhancing the resistance of crayfish to pathogens. These results suggest that early use of chemical sediment modifiers to regulate water quality and biological sediment modifiers to improve growth performance and enhance immunity may be an effective strategy in practical applications. However, the study also revealed that adding high concentrations of KMPS and PF may reduce the survival rate of crayfish, negatively affect the antioxidant and immune capacity of crayfish, and adversely affect the liver and intestinal health of crayfish. Therefore, it is critical to select the right concentration of the sediment conditioner to ensure that its positive effects are maximized and potential negative effects are minimized.

## Figures and Tables

**Table 1:** Primers used in the study.

Name	Forward Primer (5′–3′)	Reverse Primer (5′–3′)
*PcALF1*	GCTCAAGCAATAGGAGTCTCTC	CACTGGCTGGTCTCTGTTTAT
*PcALF4*	CGAGTGGTCCTTGAGTGTATG	CATCAGGCCTTCCGTATATGAG
*PcALF12*	CCCAGTGTCCTATTTGCCTTAT	CGAGCTAGATGGTGTTGAGATT
*PcCru3*	GAGCTTCTCTGCTCCAACAT	GGCTTGCATGTGTGTTGTT
*PcCru6*	GTGGTAACCTCGCAGAATGTAG	GTTTCACTGTAGGCCGATTGA
*PcCru10*	CACGTCCAGATGGTTGTAACT	CAGGTCTCACAGGAAGGTTTG
*Pc18S*	ACCGATTGAATGATTTAGTGAG	TACGGAAACCTTGTTACGAC

## Data Availability

The raw sequencing data can be found at the National Centre for Biotechnology Information (NCBI) Sequence Read Archive (SRA) with an accession number: PRJNA1248683.

## Supplementary Materials

The following supporting information can be downloaded at: https://www.mdpi.com/article/10.3390/biology14040407/s1, Figure S1: The variation of immune-related enzyme activities in the hepatopancreas of *P. clarkii* under different sediment improver treatments. Figure S2: The mRNA expression in the hepatopancreas of *P. clarkii* after supplementation with sediment improvers.

## References

[1] Subasinghe R., Soto D., Jia J. (2009). Global Aquaculture and Its Role in Sustainable Development. Rev. Aquac..

[2] Chen B., Xu X., Chen Y., Xie H., Zhang T., Mao X. (2024). Red Swamp Crayfish (*Procambarus clarkii*) as a Growing Food Source: Opportunities and Challenges in Comprehensive Research and Utilization. Foods.

[3] Peruzza L., Piazza F., Manfrin C., Bonzi L.C., Battistella S., Giulianini P.G. (2015). Reproductive Plasticity of a *Procambarus clarkii* Population Living 10 °C below Its Thermal Optimum. Aquat. Invasions.

[4] Graham Z.A., Stubbs M.B., Loughman Z.J. (2022). Digging Ability and Digging Performance in a Hyporheic Gravel-Dwelling Crayfish, the Hairy Crayfish *Cambarus friaufi* (Hobbs 1953) (Decapoda: Astacidae: Cambaridae). J. Crustac. Biol..

[5] Feng J., Pan R., Hu H.-W., Huang Q., Zheng J., Tan W., Liu Y.-R., Delgado-Baquerizo M. (2023). Effects of Integrated Rice-Crayfish Farming on Soil Biodiversity and Functions. Sci. Bull..

[6] Iber B.T., Kasan N.A. (2021). Recent Advances in Shrimp Aquaculture Wastewater Management. Heliyon.

[7] Vandecasteele B., Amery F., Ommeslag S., Vanhoutte K., Visser R., Robbens J., De Tender C., Debode J. (2021). Chemically versus Thermally Processed Brown Shrimp Shells or Chinese Mitten Crab as a Source of Chitin, Nutrients or Salts and as Microbial Stimulant in Soilless Strawberry Cultivation. Sci. Total Environ..

[8] Henares M.N.P., Medeiros M.V., Camargo A.F.M. (2020). Overview of Strategies That Contribute to the Environmental Sustainability of Pond Aquaculture: Rearing Systems, Residue Treatment, and Environmental Assessment Tools. Rev. Aquac..

[9] Hu Y., Wu G., Li R., Xiao L., Zhan X. (2020). Iron Sulphides Mediated Autotrophic Denitrification: An Emerging Bioprocess for Nitrate Pollution Mitigation and Sustainable Wastewater Treatment. Water Res..

[10] Zhang D., He J., Xu W., Li S., Liu H., Chai X. (2022). Carbon Dioxide and Methane Fluxes from Mariculture Ponds: The Potential of Sediment Improvers to Reduce Carbon Emissions. Sci. Total Environ..

[11] Hou D., Lin Z., Zhou J., Xue Y., Sun C. (2023). Germicidal Effect of Hydrogen Peroxide Nano-Silver Ion Composite Disinfectant and Its Effect on the Microbial Community of Shrimp Intestine and Rearing Water. Front. Mar. Sci..

[12] Drobac Backović D., Tokodi N. (2024). Blue Revolution Turning Green? A Global Concern of Cyanobacteria and Cyanotoxins in Freshwater Aquaculture: A Literature Review. J. Environ. Manag..

[13] Mališová E., Fašková L., Pavúková D., Híveš J., Benköová M. (2021). Removal of Cyanobacteria and Cyanotoxins by Ferrate from Polluted Lake Water. Environ. Sci. Pollut. Res..

[14] Luan Y., Wang Y., Liu C., Lv L., Xu A., Song Z. (2024). Effects of Potassium Monopersulfate on Nitrification Activity and Bacterial Community Structure of Sponge Biocarrier Biofilm in *Litopenaeus vannamei* Aquaculture System. Environ. Technol..

[15] Zhao Z., Wang B., Liu M., Jiang K., Wang L. (2022). Effects of the Non-Chlorine Oxidizer Potassium Monopersulfate on the Water Quality, Growth Performance and Microbial Community of Pacific White Shrimp (*Penaeus vannamei*) Culture Systems with Limited Water Exchange. Aquac. Res..

[16] Wang L.-G., Liu M.-Q., Xie X.-D., Sun Y.-B., Zhang M.-L., Zhao Y., Chen Q., Ding Y.-Q., Yu M.-L., Liang Z.-M. (2023). Effects of Different Water Quality Regulators on Growth Performance, Immunologic Function, and Domestic Water Quality of GIFT Tilapia. PLoS ONE.

[17] Chumpol S., Kantachote D., Nitoda T., Kanzaki H. (2018). Administration of Purple Nonsulfur Bacteria as Single Cell Protein by Mixing with Shrimp Feed to Enhance Growth, Immune Response and Survival in White Shrimp (*Litopenaeus vannamei*) Cultivation. Aquaculture.

[18] Lee S.-K., Lur H.-S., Liu C.-T. (2021). From Lab to Farm: Elucidating the Beneficial Roles of Photosynthetic Bacteria in Sustainable Agriculture. Microorganisms.

[19] Alloul A., Wille M., Lucenti P., Bossier P., Van Stappen G., Vlaeminck S.E. (2021). Purple Bacteria as Added-Value Protein Ingredient in Shrimp Feed: *Penaeus vannamei* Growth Performance, and Tolerance against *Vibrio* and Ammonia Stress. Aquaculture.

[20] Miyasaka H., Koga A., Maki T. (2023). Recent Progress in the Use of Purple Non-Sulfur Bacteria as Probiotics in Aquaculture. World J. Microbiol. Biotechnol..

[21] Li S.P., Dong J., Yang Y.B., Song Y., Liu S.C., Ai X.H. (2023). Killing effect of compound peroxymonosulfate powder on four common aquaculture pathogenic microorganisms. South China Fish. Sci..

[22] Chen Z., Li J., Zhai Q., Chang Z., Li J. (2024). Nitrogen Cycling Process and Application in Different Prawn Culture Modes. Rev. Aquac..

[23] Yue C.-F., Wang T.-T., Wang Y.-F., Peng Y. (2009). Effect of Combined Photoperiod, Water Calcium Concentration and pH on Survival, Growth, and Moulting of Juvenile Crayfish (*Procambarus clarkii*) Cultured under Laboratory Conditions. Aquac. Res..

[24] Tu J.C., Zhu M.L., Lin Z., Jiang H., Han Y.L., Yuan C., Huang S., Wang X., Chen Z. (2023). Comparative Study on the Removal Effect of Cyanobacteria by Two Algae Removal Schemes Using New Environmental Protection Oxidant. Proceedings of the 4th International Conference on Resources and Environmental Research—ICRER 2022.

[25] Zhang D.X., Liu Q., Zhang Y.M. (2014). Treating Effect of Potassium Ferrate to Aquaculture Recirculating Water. Adv. Mater. Res..

[26] Zhang X., Shu M., Wang Y., Fu L., Li W., Deng B., Liang Q., Shen W. (2014). Effect of Photosynthetic Bacteria on Water Quality and Microbiota in Grass Carp Culture. World J. Microbiol. Biotechnol..

[27] Hlordzi V., Kuebutornye F.K.A., Afriyie G., Abarike E.D., Lu Y., Chi S., Anokyewaa M.A. (2020). The Use of *Bacillus* Species in Maintenance of Water Quality in Aquaculture: A Review. Aquac. Rep..

[28] Liu J., Sun Y., Han W., Li J., Wang S., Yang Z., Cheng Y. (2022). Evaluation of the Inhibitory Effects of Four Different Microecological Preparations on Cladophora. Aquac. Int..

[29] Alvanou M.V., Feidantsis K., Staikou A., Apostolidis A.P., Michaelidis B., Giantsis I.A. (2023). For Crayfish, the Structure of the Gut Microbiota Can Serve as an Indicator of Host Health. Microorganisms.

[30] Saejung C., Chaiyarat A., Sanoamuang L. (2021). Optimization of Three Anoxygenic Photosynthetic Bacteria as Feed to Enhance Growth, Survival, and Water Quality in Fairy Shrimp (*Streptocephalus sirindhornae*) Cultivation. Aquaculture.

[31] Pedersen L.-F., Pedersen P.B., Nielsen J.L., Nielsen P.H. (2009). Peracetic Acid Degradation and Effects on Nitrification in Recirculating Aquaculture Systems. Aquaculture.

[32] Wang W., Sun J., Liu C., Xue Z. (2017). Application of Immunostimulants in Aquaculture: Current Knowledge and Future Perspectives. Aquac. Res..

[33] Patibandla S., Jiang J.-Q. Preliminary Toxicity Assessment of Pharmaceutical Solutions with and without Ferrate Treatment. Proceedings of the CEST2017 Proceedings.

[34] Zhang Y., Li Z., Kholodkevich S., Sharov A., Feng Y., Ren N., Sun K. (2019). Cadmium-Induced Oxidative Stress, Histopathology, and Transcriptome Changes in the Hepatopancreas of Freshwater Crayfish (*Procambarus clarkii*). Sci. Total Environ..

[35] Bouallegui Y. (2021). A Comprehensive Review on Crustaceans’ Immune System With a Focus on Freshwater Crayfish in Relation to Crayfish Plague Disease. Front. Immunol..

[36] Li R., Leiva C., Lemer S., Kirkendale L., Li J. (2025). Photosymbiosis Shaped Animal Genome Architecture and Gene Evolution as Revealed in Giant Clams. Commun. Biol..

[37] Li R., Zarate D., Avila-Magaña V., Li J. (2024). Comparative Transcriptomics Revealed Parallel Evolution and Innovation of Photosymbiosis Molecular Mechanisms in a Marine Bivalve. Proc. R. Soc. B.

[38] Moal V.L.-L., Servin A.L. (2006). The Front Line of Enteric Host Defense against Unwelcome Intrusion of Harmful Microorganisms: Mucins, Antimicrobial Peptides, and Microbiota. Clin. Microbiol. Rev..

[39] Rosa R.D., Barracco M.A. (2010). Antimicrobial Peptides in Crustaceans. Invertebr. Surviv. J..

[40] De la Vega E., O’Leary N.A., Shockey J.E., Robalino J., Payne C., Browdy C.L., Warr G.W., Gross P.S. (2008). Anti-Lipopolysaccharide Factor in *Litopenaeus vannamei* (*Lv*ALF): A Broad Spectrum Antimicrobial Peptide Essential for Shrimp Immunity against Bacterial and Fungal Infection. Mol. Immunol..

[41] Chelombitko M.A. (2018). Role of Reactive Oxygen Species in Inflammation: A Minireview. Mosc. Univ. Biol. Sci. Bull..

[42] Akinsemolu A.A., Onyeaka H., Odion S., Adebanjo I. (2024). Exploring *Bacillus subtilis*: Ecology, Biotechnological Applications, and Future Prospects. J. Basic Microbiol..

[43] Mu C., Zheng P., Zhao J., Wang L., Qiu L., Zhang H., Gai Y., Song L. (2011). A Novel Type III Crustin (Crus*Es*2) Identified from Chinese Mitten Crab *Eriocheir sinensis*. Fish Shellfish Immunol..

[44] Tachibana L., Telli G.S., de Carla Dias D., Gonçalves G.S., Guimarães M.C., Ishikawa C.M., Cavalcante R.B., Natori M.M., Fernandez Alarcon M.F., Tapia-Paniagua S. (2021). *Bacillus subtilis* and *Bacillus licheniformis* in Diets for Nile Tilapia (*Oreochromis niloticus*): Effects on Growth Performance, Gut Microbiota Modulation and Innate Immunology. Aquac. Res..

[45] Wang S.R., Yi L.Y., Yang H.J., Xu Q., Yuan Y.C. (2024). Effects of Glycinin on the Intestinal Microbiota and Antimicrobial-Related Genes of *Procambarus clarkii*. Acta Hydrobiol. Sin..

[46] Fang H., Wang B., Jiang K., Liu M., Wang L. (2020). Effects of *Lactobacillus pentosus* HC-2 on the Growth Performance, Intestinal Morphology, Immune-Related Genes and Intestinal Microbiota of Penaeus Vannamei Affected by Aflatoxin B1. Aquaculture.

[47] Ting-Lan Z., Yang-Fang Y.E., Chang-Kao M.U., Kai W., Rong-Hua L.I., Chun-Lin W. (2016). Gut Microbiota and Metabolic Phenotype of *Portunus trituberculatus*. Chin. J. Anal. Chem..

[48] Cao G., Qiu L., Yang G., Chen X., Wang X., Gui Y., Fan L., Meng S., Song C. (2022). Assessing the Usage Risk of the Emerging Green Chemical Potassium Ferrate in Aquaculture Environments in China: A Probabilistic Statistical Approach. J. Clean. Prod..

