# Supplementary material for: Effects of Different Sediment Improvers on the Growth Environment, Innate Immune Responses, and Intestinal Health of Procambarus clarkii
Source: Biology (Basel). 2025 Apr 11;14(4):407. doi: 10.3390/biology14040407 (PMC12025267; doi:10.3390/biology14040407)
Supplement: Supplementary file 1 [file biology-14-00407-s001.zip › biology-3551563-supplementary.pdf]

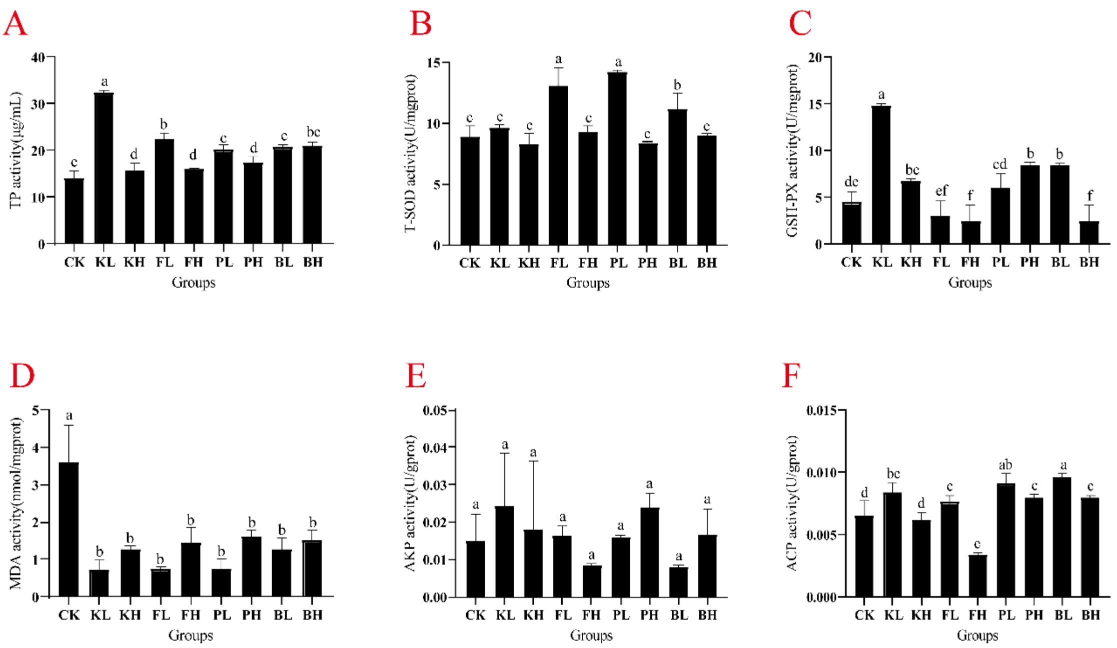

Figure S1. The variation of immune-related enzyme activities in the hepatopancreas of P. clarkii under different sediment improvers treatments. (A) Total protein (TP), (B) total superoxide dismutase (T-SOD), (C) Glutathione peroxidase (GSH-PX), (D) malondialdehyde (MDA), (E) Alkaline phosphatase (AKP) and (F) Acid phosphatase (ACP). The significant difference was determined by using a one-way analysis of variance (ANOVA), different letters indicate significantly different. Bars represent the mean ± S.D. (n = 3).

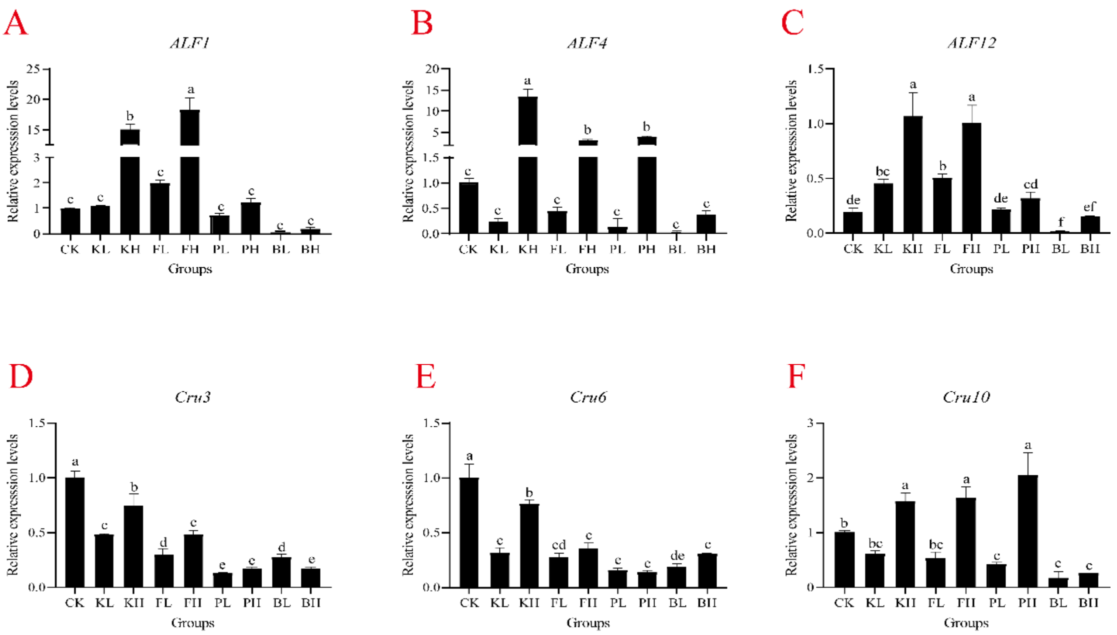

Figure S2. The mRNA expression in hepatopancreas of P. clarkii after supplementation with sediment improvers. (A) *ALF1*, (B) *ALF4*, (C) *ALF12*, (D) *Cru3*, (E) *Cru6*, and (F) *Cru10*. A one-way analysis of variance (ANOVA) was explored to calculate the significant difference, different letters indicate significantly different. Bars represent the mean ± S.D. (n = 3).
